# Application of gas flow headspace liquid phase micro extraction coupled with gas chromatography-mass spectrometry for determination of 4-methylimidazole in food samples employing experimental design optimization

**DOI:** 10.1186/s13065-022-00823-z

**Published:** 2022-05-06

**Authors:** Mahdiye Rafiei jam, Azizollah Nezhadali, Massoud Kaykhaii

**Affiliations:** 1 Department of Chemistry, Payame Noor University, P.O. Box 19395–4697, 19569 Tehran, Iran; 2 Department of Process Engineering and Chemical Technology, Faculty of Chemistry, Gdańsk University of Technology, 80-233 Gdańsk, Poland

**Keywords:** 4-Methylimidazole, Gas flow headspace liquid phase microextraction, Gas chromatography-mass spectrometry, Central composite design, Plackett–Burman design

## Abstract

**Background:**

4-Methylimidazole (4-MeI) or 4-methyl-1H-imidazole, a slightly yellowish solid with molecular formula C_4_H_6_N_2_, is a heterocyclic compound which supposedly does not exist as a natural product and is formed when carbohydrates are heating with ammonium compounds. This compound is used in pharmaceuticals, agriculture and photography chemicals, dyes and pigments, and rubber manufacturing. In the present study, a simple and efficient sample preparation method designated gas flow headspace liquid phase microextraction (GF-HS-SDME) was employed for the extraction and preconcentration of 4-methylimidazole (4-MeI) from food and beverage samples, before its determination by gas chromatography-mass spectrometry.

**Result:**

To investigate the optimal conditions for the extraction process in GF-HS-SDME method, factors affecting extraction, including selection of extraction solvent, vial volume, extraction solvent ratio, position of extracting solvent, drop volume, sample volume, stirring speed, temperature, extraction time, sample pH, ionic strength of the sample solution and gas flow rate were optimized by utilizing both one-variable-at-a-time method and Plackett–Burman design. The investigation of protocol was carried out by using a standard solution containing 100.0 μg L^−1^ of 4-MeI in deionized water.

**Conclusion:**

In this study, a simple and green analytical method based on GF-HS-SDME was proposed for the extraction and preconcentration of 4-MeI from foodstuffs, followed by GC–MS determination. The main advantage of this method is its high preconcentration factor and fastness due to the application of an inert gas stream during microextraction.

**Supplementary Information:**

The online version contains supplementary material available at 10.1186/s13065-022-00823-z.

## Background

4-Methylimidazole (4-MeI) or 4-methyl-1H-imidazole, a slightly yellowish solid with molecular formula C_4_H_6_N_2_, is a heterocyclic compound which supposedly does not exist as a natural product and is formed when carbohydrates are heating with ammonium compounds [1]. This compound is used in pharmaceuticals, agriculture and photography chemicals, dyes and pigments, and rubber manufacturing [2]. 4-MeI has been also detected in cigarette smoke [3] roasted foods and coffee [4], grilled meats and coffee [5] caramel color additives [6] and in a wide range of foods, sauces, vinegars, beers and soft drinks [7]. This contaminant compound is formed during caramel manufacturing which is in use in different foods such as cola drinks, beers, soy sauces, etc. to give a distinctive brown color to them. 4-MeI can also be formed as a byproducts during roasting in some foodstuffs [8]. This compound does not have a coordinated classification under the rules, but in more than 60% of the European Chemical Agency’s lists, 4-MeI is classified as a suspected for causing cancer, in addition, it is reported as a neurotoxic [9] and International Agency for Research on Cancer classifies 4-MeI as a “potentially carcinogenic to humans” [10]. Mice exposed to the high levels of 4-MeI, reported to have more lung tumors [11]. Due to the negative evaluation, the European Food & Safety Authority has suggested a no observed adverse effect level of 80 mg kg^−1^ day^−1^ for 4-MeI. The World Health Organization and the European Union for the caramel class E150c and E150d, set 250 mg kg^−1^ as the maximum limit of 4-MeI in caramel [9]. As a result, reliable analytical methods are needed detect exact concentration of 4-MeI in foodstuffs. Methods such as gas chromatography (GC) [4], capillary electrophoresis [12], liquid chromatography (LC) [6], and heart cutting two dimensional LC have been reported for the identification and quantification of 4-MeI, so far. However, these methods need laborious and time consuming sample preparation pre-treatment of sample and are not very sensitive for the analysis of 4-MeI due to its special characteristics such as low volatility, low molecular weight, high polarity and lack of sufficiently strong chromophore group in its chemical structure [13]. Lately, more sensitive methods basing on gas chromatography coupled with mass spectrometry detector (GC–MS) [14, 15], have been presented. An advantage associated to gas chromatography-mass spectrometry (GC–MS) method includes shorter sample preparation time, detection at low levels with high selectivity. While chromatography has the power of separating signals of matrix components from the analyte, the main drawbacks associated with this technique is unfeasibility of detecting low concentrations of analytes [16]. This problem can be overcome by applying a proper extraction technique prior to performing chromatography. Techniques such as solid phase extraction [17–20], solid-phase micro-extraction (SPME) [21] dispersive micro-solid phase extraction [22], supercritical fluid extraction [23], cation exchange sorbents [24], enzyme-linked immunosorbent assay [25] and ion-pair extraction and derivatization [26, 27] are excellent sample preparation techniques that are successfully employed to achieve this goal for the extraction and preconcentration of 4-MeI; however, they each require a specialized apparatus with some type of solid or polymeric sorbent to collect the analyte. For example, the main drawbacks of SPME are that its fibers are expensive and have a limited lifetime, as they tend to degrade with increased usage [16]. The prototype of single-drop microextraction (SDME) was developed by Jeannot and Cantwell [28, 29] and has been recognized as one of the simplest miniaturized sample preparation tools for the isolation and preconcentration of many analytes from complicated sample matrices [30]. SDME which was the first liquid–liquid microextraction technique initially implemented in direct immersion mode and later in headspace (HS) mode for volatile analytes [30]. Headspace single-drop microextraction (HS-SDME) was introduced by Theis et al. in 2001 [31]; in which, analytes are evaporated from the solid or aqueous sample into the gas phase and then enrich to a micro-liter solvent drop hanging from a micro-syringe tip placed in the HS of sample. This method has many advantages such as convenience, very low extraction solvent consumption, fastness and ability of being automated. As a result, HS-SDME has been used for the determination of volatile analytes such as benzene hydrocarbon compounds [32], aliphatic amines [33], phenols [34], chlorinated anilines [35] poly nuclear aromatic hydrocarbons (PAHs) [36] and so on. In this regards, two simple and efficient techniques of gas flow headspace liquid phase microextraction [37] and gas purge microsyringe extraction (GPMSE) [37] has been developed for the extraction and determination of PAHs, organochlorine pesticides and alkylphenols. In GPMSE, by using a gas flowing system, temperature control and a conventional microsyringe, the surface area of the liquid phase micro-solvent could be greatly enhanced, as a result fast kinetic of extraction (2 min extraction) and high enrichment factors obtained. A comprehensive list can be found in a very recent review article published by Kailasa, et al. [30]. This technique has never been used for the extraction of 4-MeI, however, due to low vapor pressure of this compound [21]. Here, we applied a stream of nitrogen gas to pass throughout the headspace of the samples during the extraction period to facilitate diffusion of the analyte to the HS of the sample even at lower temperatures. This was also increased the preconcentration factor and reduced the extraction time; Also, a new and environmentally friendly method was used to determine 4-MeI in drinks and food products. In order to find the best extraction conditions, first, Plackett–Burman design (PBD) was employed for screening of insignificant factors and then, central composite design (CCD) was employed to optimize parameters affecting microextraction.

## Experimental

### Chemical and materials

4-methylimidazole (purity > 98%), 1-methylimidazole (1-MeI) and glycerol (GLY) were purchased from Sigma-Aldrich Chemie GmbH (Steinheim, Germany). Ethylene glycol (EG), sodium chloride (NaCl), sodium hydroxide (NaOH), hydrochloric acid (HCl) and all other reagents (analytical grade) were purchased from Merck. Nitrogen gas (Sabalan Co., Tehran, Iran) with 99.999% purity was used for gas flow of GF-HS-SDME system. Stock solution of 4-MeI and 1-MeI were prepared by dissolving 1.0 g of each compound separately in 1 L of ethanol to obtain a concentration of 1000.0 mg L^−1^. All solutions were kept at 4 °C in a dark place when not in use, being stable for 3 months [21].

### Instrumentation and software

The determination of 4-MeI was performed on an Agilent gas chromatograph (GC) 6890 (USA) equipped with an electronically controlled split/splitless inlet. A 5973 series mass selective detector (MSD) with electron impact (EI, 70 eV) ionization chamber was used for mass spectrometry. The gas chromatography separation was conducted with a DB-5 MS fused-silca capillary column containing 5% diphenyl and 95% dimethylpolysiloxane (60 m × 0.25 mm i.d., 0.25 µm film thickness). Flow rate of Helium (purity of 99.999%) was set to 1.0 mL min^−1^. The injection was made in splitless mode and temperature was operated at 280 °C. The temperature program was set as: 80 °C held for 1 min, ramped to 280 °C at 20 °C min^−1^ and held for 3 min with a total run time of 12.5 min and the MS transfer line temperature was 300 °C. Mass spectrometric parameters were set as follows: electron ionization (EI) with 70 eV energy; mass ion source temperature was kept at 230 °C; quadrupole temperature was set at 150 °C and the solvent delay was set at 5 min for all analyses. 4-MeI was identified by comparing the mass spectrum obtained for analyte to that of the reference compound in the instrument's library using the US National Institute of Standard and Technology (NIST) libraries search. 4-MeI were determined in the selected ion monitoring (SIM) mode. A target ion (most abundance ion) with m/z of 182 and three qualifier ions with m/z of 109, 81 and 82 were monitored for the target analyte and for 1-MeI ions with m/z 82, 81 and 54 were selected. The retention time of the target ion and the qualifier-to-target ion ratios for 4-MeI as confirmation of identification. Agilent Chemstation software was used for data processing/reporting and GC–MS control. For GC analysis a microsyringe (10.0 μL, Hamilton, Switzerland) was used and vials with screw silicone septa (Chromacol, Trumbull, CT) were employed.

The Placket-Burman (P-B) and central composite design (CCD) matrices were estimated with MINITAB 16 statistical software package (Minitab Inc., State College, USA).

### Real samples preparation

Caramel colored drinks (cola and energy drinks) and foodstuffs (coffee, instant coffee, caramel and cola extract) were purchased from the local markets at Zahedan, Iran and Tara Teb Tajhiz Co., Tehran, Iran. All samples were stored at room temperature on their original packages. For carbonated drinks, an aliquot of 100.0 mL was degassed by ultrasonication in a bath (JK-DUC-2200LHC, JKI, China) for 30 min before sample pretreatment. 1.0 mL of each liquid sample was diluted to 10.0 mL with H_2_O and 8.0 mL of the solution was taken for extraction. Solid samples were thoroughly homogenized with a laboratory blender and grinded to pass a 25 mesh sieve. 1.0 g of grinded samples were transferred into 10.0 mL beakers, dissolved or soaked in 2.0 mL of H_2_O and then 8.0 mL methanol was added. Solutions were agitated on a shaker for 5 min. To perform extraction, 8.0 mL of either liquid or dissolved sample aliquots and 8.0 μL of 50.0 mg L^−1^ of 1-MeI as internal standard were placed in 20.0 mL carrier-lined screw caps vials and 4.0 g NaCl was dissolved and their pH was adjusted to 10.0 by drop-wise addition of either 0.1 M HCl or 0.1 M KOH.

### GF-HS-SDME procedure

**Fig. 1:**
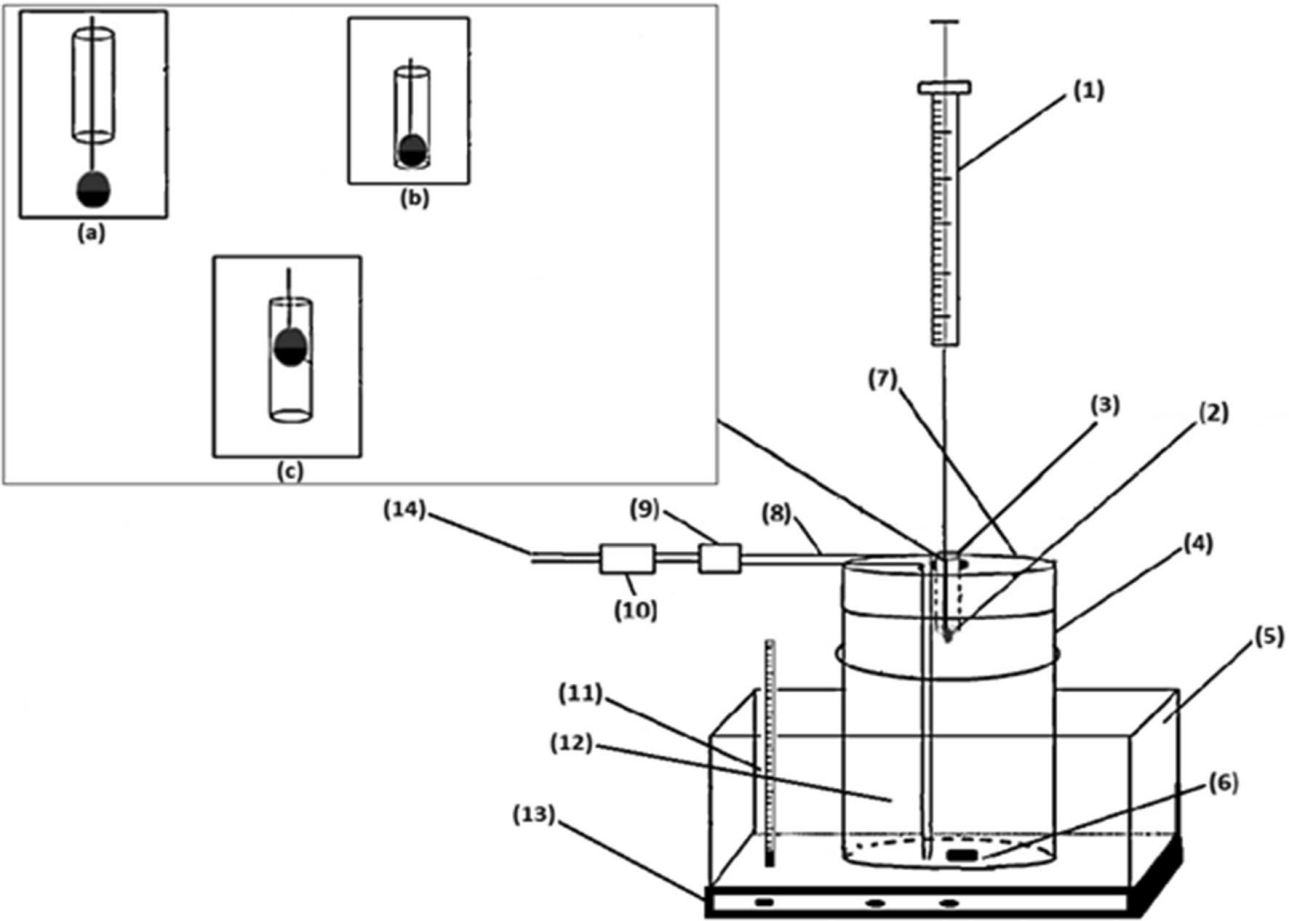
Schematic of the GF-HS-SDME apparatus designed and used in this research. **1** 10 µL microsyringe, **2** Extraction solvent, **3** N_2_ Gas outlet, **4** Sample vial, **5** Water bath, **6** Stir bar, **7** PTFE lined cap, **8** PTFE line, **9** Digital flow meter, **10** Gas flow controller, **11** Thermometer, **12** Sample, **13** Heater magnetic stirrer, **14** N_2_ Gas inlet. Insert shows positions of the microdrop of the extracting solvent in the N_2_ gas outlet **a** 0.5 cm out of the bottom, **b** at the bottom of the channel, **c** 0.5 cm inside the channel

Figure 1 shows the apparatus which was designed and applied for GF-HS-SDME in this study. The apparatus was consisted of a gas inlet flow line, a sample vial, a water bath on a magnetic stirrer, a thermometer and a 10.0 µL microsyringe. For flowing nitrogen gas inside the vial, a PTFE tubing [8] was inserted in a way to reach to the bottom of the sample vial for nitrogen gas entrance.

After purging sample and passing around the microdrop, it could exit through the glass tube [1], (id = 1.8 mm) which was inserted into the middle of sample vial cap. In this way, while the N_2_ gas passing through the sample, it can agitate it and at the same time it can purge volatile components into the headspace of the sample. Narrow tube 1 ensures that all purged components are in contact with the microdrop before exit of the entrainer gas. A digital gas flow controller [10] was employed for precise controlling of N_2_ flow throughout the sample vial.

Before each extraction, microsyringe was washed thoroughly several times with dichloromethane then with hexane and at least ten times with the extracting solvent to be sure of the absence of air bubbles. During extraction, the samples were stirred using a stirrer (model F20520162, VELP (Spain)) with a Teflon-coated magnetic stir bar.

The extraction process consisted of the following steps: (i) 2.5 μL of extraction solvent was withdrawn into the microsyringe. (ii) The microsyringe needle was passed through the septum of extraction vial and the needle was kept above the surface of the liquid sample. (iii) The plunger was pressed so that the extraction solvent was suspended very close to the surface of the sample, and held for 20 min. (iv) After extraction is completed, the plunger was withdrawn back and the microdrop was retracted back into the microsyringe.

The N_2_ flow was stopped and the syringe was removed from the top the surface of the liquid sample. After the extraction time, the needle was removed from the vial sample and the 4-MeI-enriched organic solvent was injected into the GC/MS for analysis. The same process was repeated at least 3 times. The precision of the method was improved by positioning the needle in the headspace of the sample at a fixed length with stands and clamps. To perform extraction, sample was put in a sample vial and 2.5 μL of glycerol:ethylene glycol (62:38) was withdrawn into a micro-syringe and the needle of the microsyringe was inserted into the silicon lined cap of the vial. The needle tip was always located in a fixed position inside the gas outlet channel. The plunger was pressed to hang 2.5 μL solvent drop from the tip of the needle. The solution was stirred at the rate of 100 rpm during extraction. Temperature and flow of the N_2_ gas were set at 65.0 °C and 2 mL min^−1^, respectively. After extraction, the solvent drop containing the extracted analyte was retracted back into the syringe and injected to GC–MS. Peak area of the peaks was used for quantification and each experiment was performed at least in triplicate.

## Results and discussion

### GF-HS-SDME optimization

To investigate the optimal conditions for the extraction process in GF-HS-SDME method, factors affecting extraction, including selection of extraction solvent, vial volume, extraction solvent ratio, position of extracting solvent, drop volume, sample volume, stirring speed, temperature, extraction time, sample pH, ionic strength of the sample solution and gas flow rate were optimized by utilizing both one-variable-at-a-time method and PBD. The investigation of protocol was carried out by using a standard solution containing 100.0 μg L^−1^ of 4-MeI in deionized water.

### One-factor-at-a-time optimization

Due to limitations of PBD for optimization of parameters such as selection of extraction solvent, vial volume and position of the microdrop of extracting solvent in the gas outlet channel, these parameters were optimized by one-factor-at-a-time method, as indicated below.

### Type of extraction solvent

**Fig. 2:**
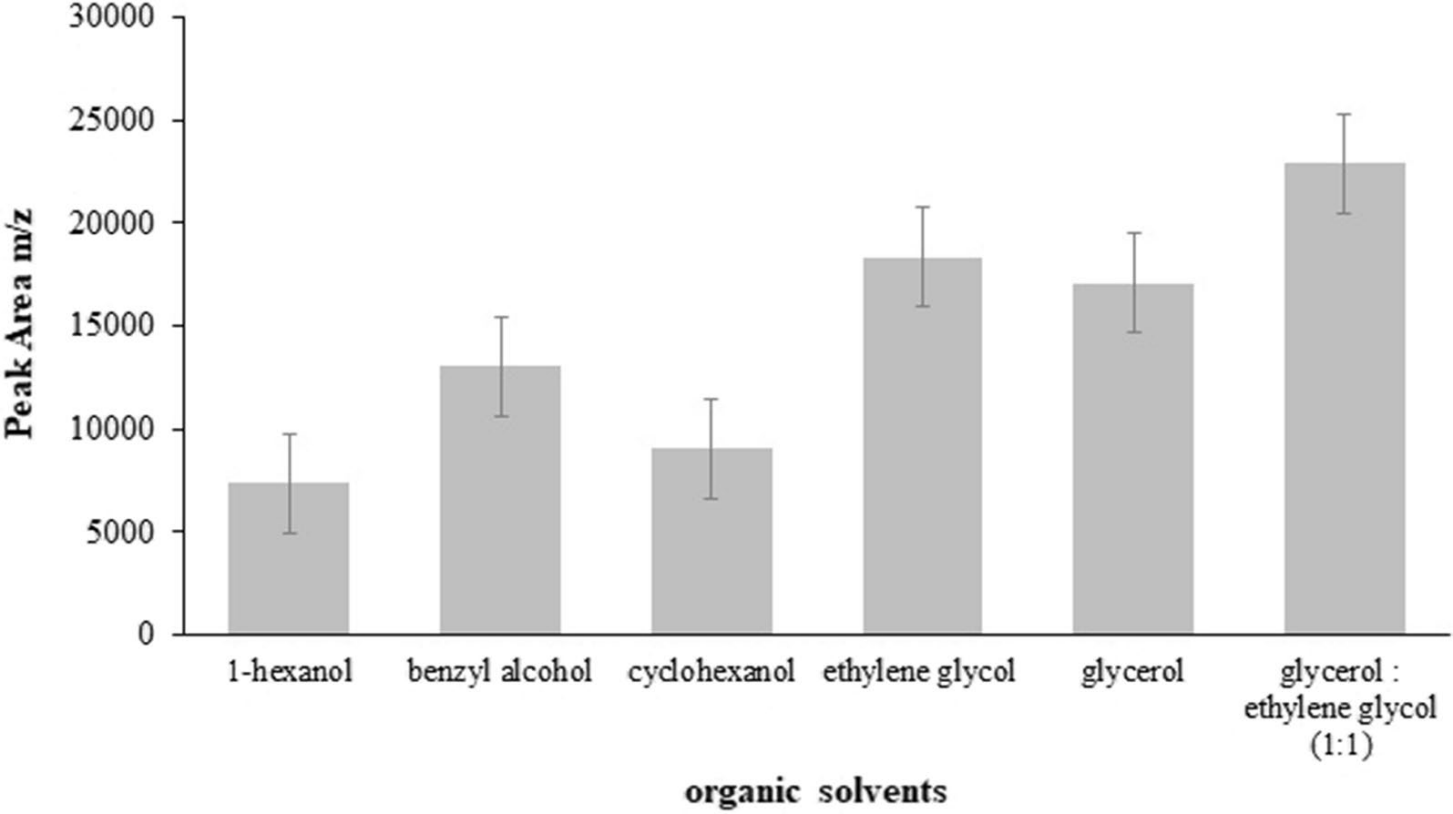
Effect of different organic solvents on the extraction efficiency (sample volume 2.0 mL, temperature 110.0 °C; extraction time 45 min; 1.0 g of NaCl; gas flow rate 3.0 mL min^−1^; stirring speed 200 rpm; drop volume 2.0 μL; vial volume 10.0 mL; n = 3)

The type of organic solvent used for extracting analyte is an important factor in all SDME methods. The solvent should have a good affinity for the analyte, low solubility in water, be stable during the extraction process, and have good chromatographic behavior [38]. In this research, different commonly solvents such as 1-hexanol, benzyl alcohol, cyclohexaol, ethylene glycol, glycerol and mixture of 1:1 (v/v) glycerol:ethylene glycol were utilized to optimize the effect of extracting solvent on the percent of extraction. As indicated in Fig. 2, a 1:1 (v/v) mixture of glycerol:ethylene glycol was showed the best extraction efficiency for extracting analyte.

### Effect of vial volume

**Fig. 3:**
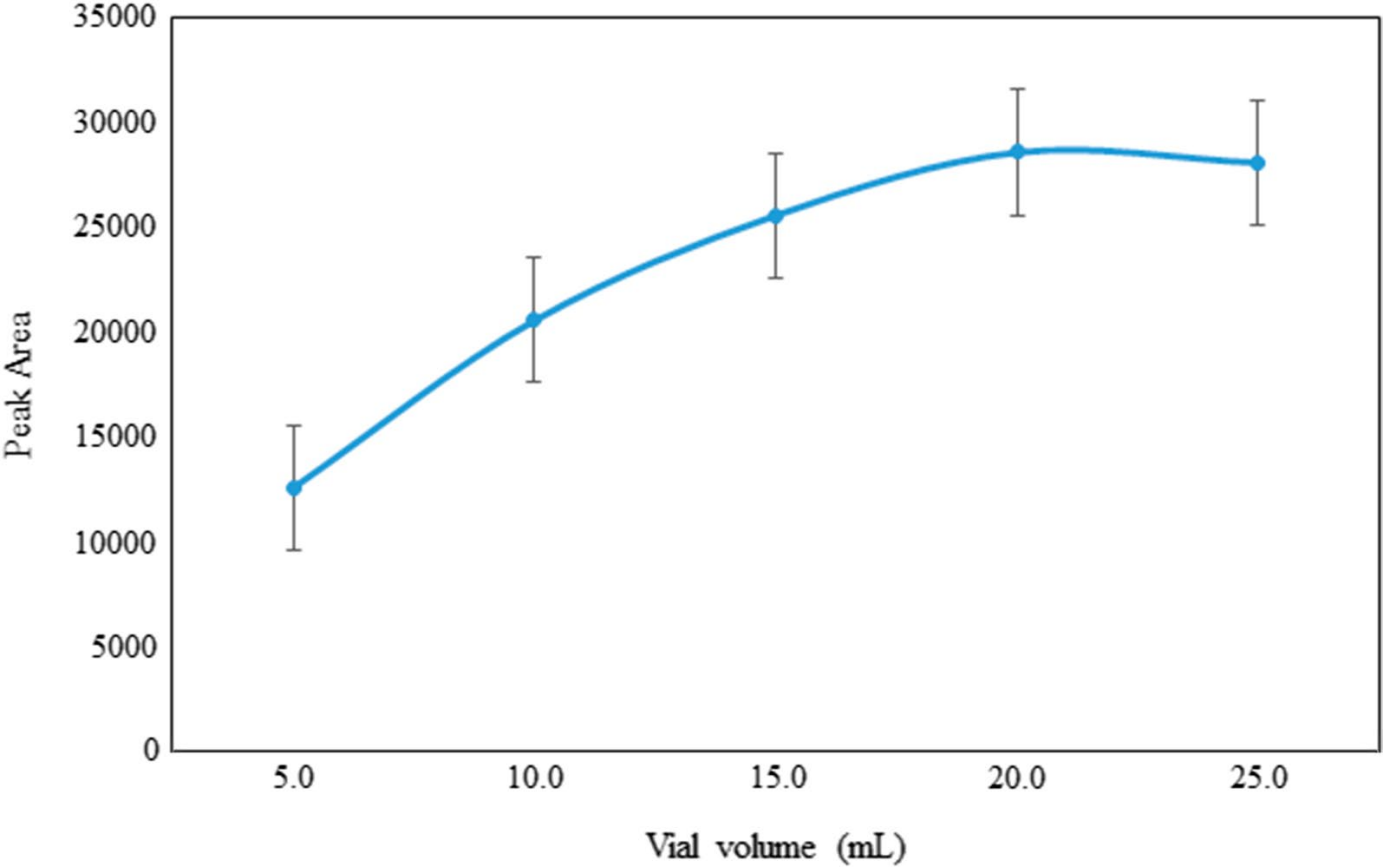
Effect of vial volume on the extraction efficiency (sample volume 2.0 mL; temperature 110.0 °C; extraction time 45 min; 1.0 g of NaCl; gas flow rate 3.0 mL min^−1^; string speed 200 rpm; drop volume 2.0 μL; extracting solvent, glycerin:ethylene glycol (1:1); n = 3)

Based on the ideal gas equation, it can be predicted that the amount of a chemical in the gas phase increases with increasing gas volume under a given temperature and pressure. In order to confirm this prediction, preliminary experiments on the relationship between the enrichment factor and the gas volume were carried out with the GF-HS-SDME technique. Sample vial volumes of 10.0, 15.0, 20.0 and 25.0 mL were selected and 2.0 mL of 100.0 μg L^−1^ of 4-MeI solution were poured in them after adjustment of temperature at 110 °C and gas flow rate 3.0 mL min^−1^, was carried out by following the general GF-HS-SDME and results are depicted in Fig. 3 for comparison. It was found that peak areas were dramatically increased by increasing sample vial volume. It was also found that the factors by which the relative areas increased were nearly equal to the ratio of the corresponding volumes. Based on the Fig. 3. 20.0 mL had the best performance for vial volume. Based on the Fig. 3, after vial volumes of 20.0 mL, the relative area decreased, so the sample vial volumes of 20.0 mL was selected for further experiments.

### Effect of position of the hanging droplet

During experiments, it was found that the position of the hanging droplet greatly affects the enrichment factor of the method. Therefore, the effect of the position of it in the gas outlet channel was studied. Three different locations were selected (Fig. 1): 0.5 cm out of the bottom of the channel; at the bottom of the channel, and 0.5 cm inside the channel form the bottom side. It was found that when micro-drop is positioned at the bottom of the channel, signal increases several times. That’s because almost all of the flowing gas passed all the way through the surface of the extracting solvent and so analyte vapor can better interact with the solvent.

### Placket–Burman design

After optimization of the above mentioned parameters with one-factor-at-a-time method, PBD was applied as a screening to select remaining most statistically significant parameters for further optimization. This design can identify influence factors the GF-HS-SDME, 4-MeI extraction and GC–MS measurement processes. It does not represent the exact value, but it does provide valuable information about each variable with a relatively limited experimental performance [39, 40]. A Plackett–Burman type (III) resolution scheme was performed for 9 factors, included of 12 runs, it is done repeatedly to, to annul the effects of external variables [41]. The factors and level of variables level (low and high respectively) was chose to coat the range of optimized conditions that was guessed utilizing the one-factor-at-a-time procedure (Table 1). Table 2 displays the statistical evaluation of the outcomes generated standard for each run. The most important effect of each factor was approximated employing minimum square regression which shows the importance relative to the reply (total chromatographic peak area, 4-MeI).

**Table 1:** The experimental field definition for PBD

Variable	Symbol	Low ( −)	High ( +)
Micro-drop volume (µL)	Drop volume	2.0	5.0
Sample volume (mL)	Sample volume	1.0	8.0
Stirring speed (rpm)	Stirring speed	100	1200
Extraction temperature (^o^C)	Temp	60.0	120.0
Extraction time (min)	Time	20	60
pH	pH	4.0	10.0
Ionic strength (g mL^−1^)	Salt	0.0	0.5
Gas flow (mL min^−1^)	N2	0.0	4.5
Extraction solvent ratio (%)	GLY:EG	Glycerol	Ethylene glycol

**Table 2:** The results of Plackett–Burman experimental design matrix

Run Order	Drop Volume (µL)	Sample Volume (mL)	Stirring Speed (rpm)	Temperature (^o^C)	Time (min)	pH	Salt (g mL^−1^)	N_2_ (mL min^−1^)	GLY:EG (%)
1	5.0	8.0	1200	60.0	60	10.0	0.0	4.5	Glycerol
2	5.0	1.0	100	60.0	60	10.0	0.5	0.0	Ethylene glycol
3	5.0	8.0	100	120.0	60	4.0	0.5	0.0	Glycerol
4	2.0	1.0	1200	120.0	60	4.0	0.5	4.5	Glycerol
5	2.0	8.0	1200	120.0	20	10.0	0.5	0.0	Ethylene glycol
6	5.0	1.0	1200	60.0	20	4.0	0.5	4.5	Ethylene glycol
7	5.0	8.0	100	120.0	20	4.0	0.0	4.5	Ethylene glycol
8	2.0	1.0	100	60.0	20	4.0	0.0	0.0	Glycerol
9	2.0	8.0	100	60.0	20	10.0	0.5	4.5	Glycerol
10	2.0	8.0	1200	60.0	60	4.0	0.0	0.0	Ethylene glycol
11	5.0	1.0	1200	120.0	20	10.0	0.0	0.0	Glycerol
12	2.0	1.0	100	120.0	60	10.0	0.0	4.5	Ethylene glycol

**Fig. 4:**
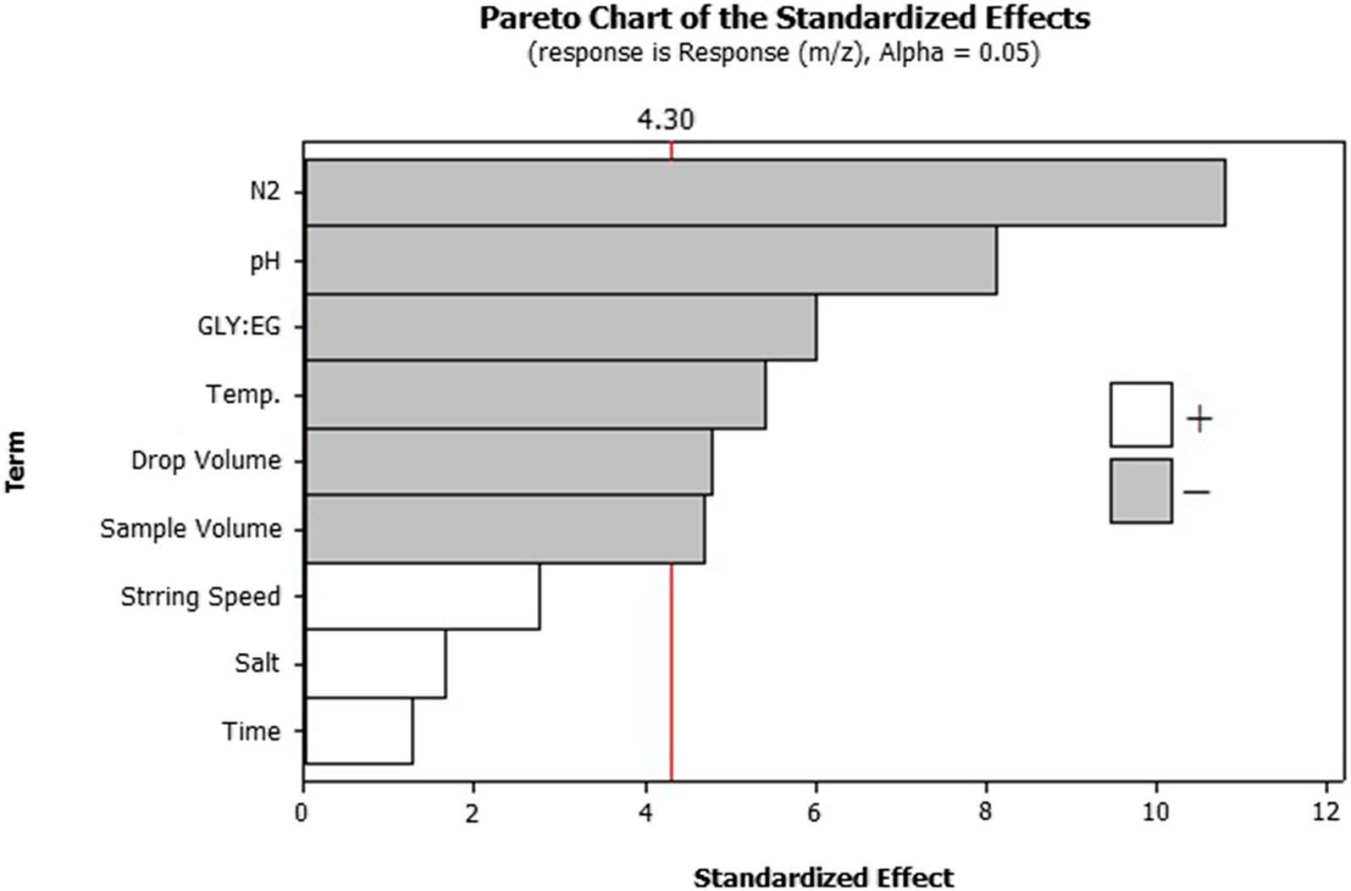
The standardized main effect Pareto chart for PBD

As shown in Fig. 4, the length of the bar in the Pareto chart is comparative to the conclusive value of the chief effect [38, 39] and recommended a least amount *t*-value of 4.30 at a certainty level of 95.0%. Lengths are comparative to the absolute values of approximated effects and t-value is involved reference line as a vertical.

**Fig. 5:**
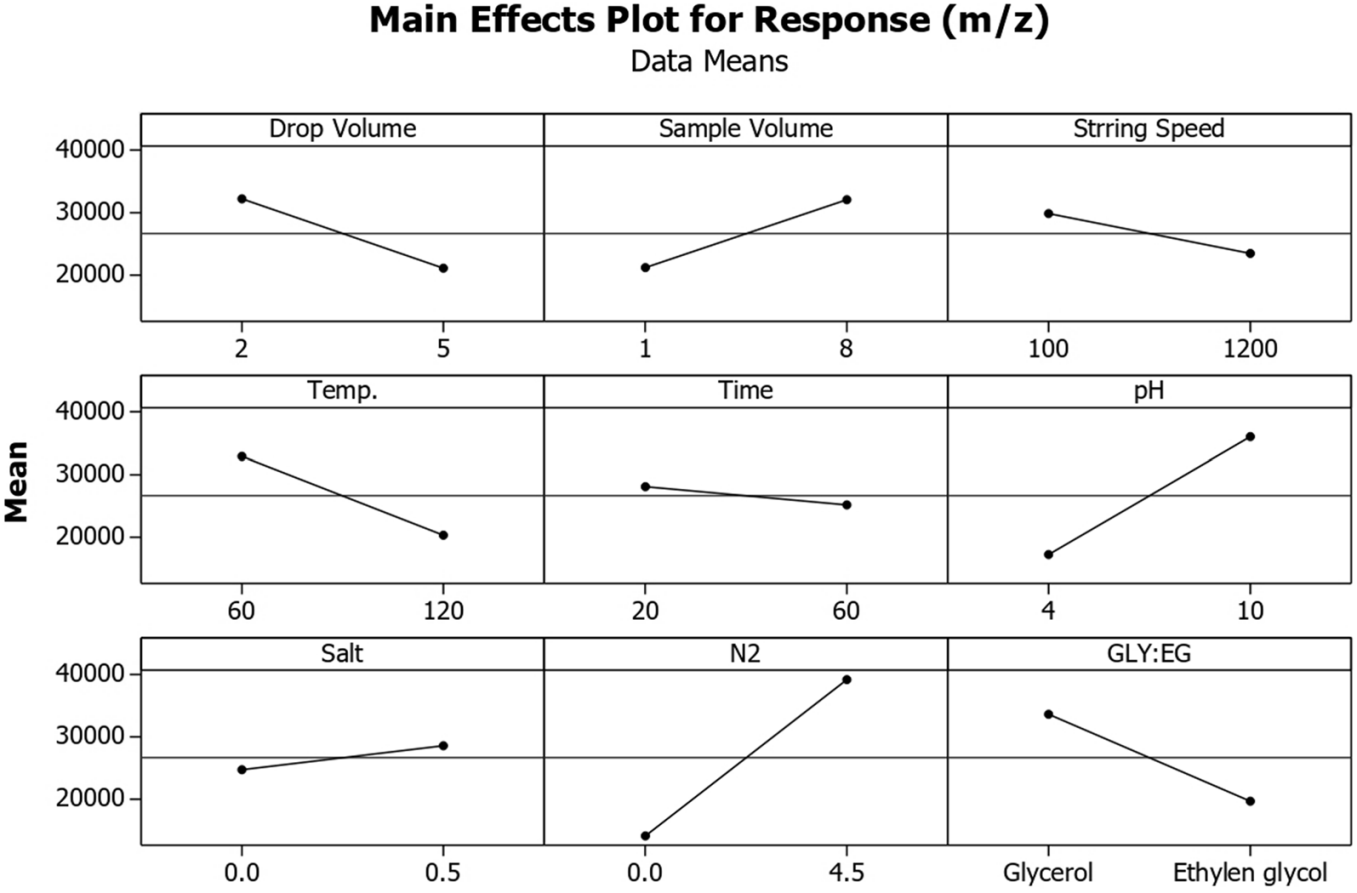
The main effect plot for PBD

The unfixed were investigated as numerically imperative parameters. In addition, the positive and negative signals (along the lines of a white and gray bar filling, respectively) demonstrate that whether the response would be improved from the low to high level or not. The main effect plot (Fig. 5), when sample volumes, pH and inert gas flow rate, increase from low to high values, the extraction efficiency also increases, and the extraction efficiency increases with decrease in micro-drop volume and extraction temperature.

Other factors such as stirring rate of solution, ionic strength in sample solution and extraction time showed no significant effect, and thus could be kept fixed at any value, since their adjustment will have equal effect on the extraction efficiency. Therefore, for the optimization step, all other factors were fixed, while sample volumes, pH, inert gas flow rate micro-drop volume and extraction temperature were considered for further optimization.

### Central composite design

Based on the results of the screening design, an optimization procedure was planned. Six variables including inert gas flow (N_2_), pH of sample (pH), ratio of components of the extracting solvent (GLY:EG), extraction temperature (Temp), micro-drop volume (drop volume) and sample volume were simultaneously optimized using CCD and the effects as well as their mutual interactions were studied. A three-level CCD with 53 runs was carried out for optimization. The significant variables involved in the generation of CCD, their levels and the plan matrix are utilized in an additional Table (Additional file 1: Table S1).

To discovering the most suitable fit with the experimental information, an answer surface model was expanded employing the regression analysis by considering dissimilar mixtures of the linear, squared and interface terms in polynomial equations. The Kolmogorov–Smirnov test was applied to the obtained data, and the results showed that the determined 4-MeI in the food samples followed a normal distribution with a significance level of 0.05. The adequacy of each model was considered employing the analysis of variance (ANOVA) and a high p-value for lack-of-fit (LOF) of 0.66 was achieved for the following equation proposing that the quadratic model was important. By asking the regression analysis, the following equation was achieved based on the regression analysis for the GF-HS-SDME method.

Area (m/z) =  − 2,084,719 – 501,754 × Drop volume + 79,009.9 × Sample volume + 4193.11 × Temp. + 332,381 × pH – 342,120 × N_2_ + 39,946.0 × GLY:EG + 109,381 × (Drop volume)^2^ − 5338.01 × (Sample volume)^2^ − 47.7186 × (Temp.)^2^ − 17,237.0 × (pH)^2^ + 52,414.9 × (N_2_)^2^ − 301.883 × (GLY:EG)^2^ − 883.736 × (Drop volume) (Sample volume) + 853.610 × (Drop volume) (Temp.) + 5723.94 × (Drop volume) (pH) + 4818.65 × (Drop volume) (N_2_) − 512.931 × (Drop volume) (GLY:EG) + 6.91736 × (Sample volume) (Temp.) − 113.725 × (Sample volume) (pH) + 865.950 × (Sample volume)(pH) + 101.198 × (Sample volume) (GLY:EG) + 108.561 × (Temp.) (pH) + 248.381 × (Temp.) (N_2_) − 28.7939 × (Temp.) (GLY:EG) − 204.625 × (pH) (N_2_) + 105.028 × (pH) (GLY:EG) − 622.353 × (N2)( GLY:EG).

Where “Area” is the response of GC–MS to 4-MeI values and Drop volume, Sample volume, Temp., pH, N_2_ and GLY:EG are the real costs of the important factors. With the intention of attain the maximum reply, the achieved equation was calculated and the parameters of drop volume, sample volume, temp., pH, N_2_ and GLY:EG were computed to be 2.5 µL, 8.0 mL, 65.0 °C, 10.0, 2.0 mL min^−1^ and 62:38 respectively. Furthermore, ANOVA and regression analyses were performed to investigate the effects of changeable and their interactive effects. Determination of statistical significance was investigated at a level of p < 0.05. The lack of fit p-value was achieved 0.778. As illustrated in Additional file 1: Table S1, the relative area enhanced with an enhance in the transporter gas flow rate up to 2.0 mL min^−1^, this may be for the reason that the rate of mass transfer of the target compound raised with the improve in the gas flow rate. When the gas flow rate was higher than 2 mL min^−1^, the comparative area reduced, this is perhaps due to fast gas flow rate can diminish the opportunity of contact between the target compound and the extracting solvent [41]. Consistent with the outcomes of the Placket-Burman plan and the CCD, at high temperatures, the mass transfer and diffusion of the analyte in the direction of solvent drop is speedy. The removal temperature is under the influence both the kinetics and thermodynamics of the headspace extraction methods of volatile and semi-volatile compounds [37]. Generally, an enhancement in the temperature is come with by higher Henry constants and diffusion coefficients, which products better extraction in a HS mode. Conversely, the sorption procedure on the drop is exothermic; therefore, higher temperatures reduce the partition coefficients of the analytes [26]. Also, higher temperatures can make the micro-drop variable and depreciate procedure repeatability [42, 43].

The extraction temperature in HS-SDME cannot be studied exclusively, without considering the extraction time. To study the effect of temperature on extraction yield, temperatures of 30.0, 60.0 and 90.0 °C were investigated by CCD. Considering the Additional file 1: Table S1 data, 65.0 °C had the best performance for analyte extraction, and consequently, was chosen as the optimum extraction temperature. It is evident that increasing the ionic strength of a solution usually promotes transferring of organic molecules to the head space and hence to the extracting droplet (salting-out effect) [44]. On the extraction efficiency, in step PBD of NaCl 0.0 and 0.5 g mL^−1^ was added to sample solution of 4-MeI with a concentration of 100.0 μg L^−1^ were studied and show no significant effect. According to the results (Fig. 5), the amount of NaCl (0.5 g mL^−1^) yielded the best performance.

Similar to any other microextraction method, GF-HS-SDME is an equilibrium-based process [45]. For optimum repeatability of the analysis, it is necessary to find a time in which equilibrium between the extracting liquid and the headspace reaches. Therefore, in PBD, times of 60 and 20 min were examined. The results showed (Fig. 5) that the maximum extraction was obtained in 20 min, and show no significant effect on the extraction yield. It was also noticed that because of hydrophobicity of ethylene glycol, after 40 min, the volume of the micro-drop grew up from 2.5 to 4.0 µL, which causes its detachment from the needle, so 20 min was set as the extraction time.

Sampling agitation enhances extraction and reduces extraction time because the equilibrium between the aqueous and vapor phases can be reached faster [46]. Furthermore, convection is induced in the headspace when the aqueous phase is efficiently stirred [34]. However, increasing the stirring speed, causes spattering of the solution which contaminates the extracting drop, and also induced vibration which destabilizes it. In suggested GF-HS-SDME method, since high amounts of the analytes is accessible to the droplet by passing the gas flow during the extraction process, it is expected that stirring rate has no significant effect on the extraction. In step PBD, stirring speeds of 100 and 1200 rpm were examined, as shown in Fig. 5, the 100 rpm, so that this provides higher enrichment factors than 1200 rpm, so 100 rpm was chosen as the optimum stirring speed.

Volume of the micro-drop is another important factor for having an efficient extraction. In general, increasing the drop volume results in the significant improvement of the extraction efficiency. However, when the microdrop volume increases further, the drop became unstable due to the gravity and falls off. [47]. Based on the data shown in Additional file 1: Table S1, 2.5 µL micro-drop had the best performance and selected for further experiments. Sample volume is another important parameter affecting SDME techniques. A better equilibrium between the sample and its headspace can be achieved by selecting the appropriate sample volume. In this study, in order to have an adequate headspace volume to introduce the syringe and form the microdrop, the sample volume was fixed to 8.0 mL.

### Method performance and validation

The validation of analytical methodology has been observed to be a quality assurance step in method development [48] used to confirm the method performance and its suitability for the intended purpose. Here, the figures of merit of the developed method was validated in terms of linearity, accuracy (reported as the relative recovery), intra-day and inter-day precisions, limit of detection (LOD) and limit of quantification (LOQ), using the optimized GF-HS-SDME parameters.

### Linearity, LOD, LOQ and Enrichment factor

The linearity of the proposed method was evaluated using matrix-matched calibration curves. All samples were spiked with standard working solutions at eight concentration levels ranging from 2.9 to 300.0 μg L^−1^ and 50.0 μg L^−1^ of 1-MeI and were pretreated according to the GF-HS-SDME method described previously, and each experiment was performed at least in triplicate. The ratio of the peak area of the standard to that of the internal standard was used to quantify the analyte. The determination coefficients (R^2^) of the matrix-matched calibration curves were found as higher than 0.998. So, it can be regarded that the matrix-matched standard calibration curves shows good linearity for 4-MeI in the mentioned matrices in the studied range. LOD (1.1 μg L^−1^) and LOQ (2.9 μg L^−1^) values were calculated experimentally at a signal-to-noise ratio of 3 and 10, respectively, using the standard deviation of the y-intercept of the regression line of the calibration curve. Enrichment factor (EF) was determined from the analyte concentration ratio in the GLY: EG phase to the water solution and was in the 58.5 for the 4-MeI determination with a concentration of 100.0 μg L^−1^. These results indicated that the GF-HS-SDME-GC/MS procedure has high enrichment factors, low LOQ and LOD, proper relative standard deviation (RSD), and wide linearity for the 4-MeI determination.

### Accuracy and precision

The intra-day precision was estimated by performing three extractions of each of concentration levels of 10, 100 and 200 µg L^−1^ of 4-MeI in a single day; and inter-day precision was estimated based on three extractions per day for 3 days for the aforementioned concentrations. The obtained RSD values were in the range of 0.2 to 3.4% (Table 3). To validate the method and evaluate the effect of the matrix, all samples were spiked with 4-MeI. Relative recoveries of 4-MeI spiked samples at three different concentration levels (n = 3), ranging from 85.3 to 93.0% (Table 3). These results demonstrate that the sample matrices, in our present context, have no significant effect on GF-HS-SDME for determination of 4-MeI.

**Table 3:** Accuracy, Intra and inter day precisions for GF-HS-SDME of 4-MeI extraction in real samples (mean ± SD^a^, n = 3)

Sample	Amount measured in sample (µg L^−1^)	4-MeI added (µg L^−1^)	Intra-day precision		Inter-day precision	
			4-MeI found (µg L^−1^ ± SD)	Recovery (RSD%)	4-MeI found (µg L^−1^ ± SD)	Recovery (RSD%)
Distilled water	Not Detected	10.0	8.7 ± 0.5	86.7 (0.6)	9.3 ± 0.3	93.0 (0.4)
		100.0	91.3 ± 2.0	91.3 (2.2)	90.6 ± 1.5	90.6 (1.6)
		200.0	179.6 ± 1.4	89.8 (1.5)	176.3 ± 2.7	88.1 (3.0)
Energy drink-1	Not Detected	10.0	8.7 ± 0.2	86.7 (0.2)	9.1 ± 0.8	90.7 (0.9)
		100.0	87.0 ± 2.0	87.0 (2.3)	85.2 ± 1.2	85.2 (1.4)
		200.0	184.8 ± 1.3	92.4 (1.4)	182.4 ± 2.6	91.2 (2.9)
Energy drink-2	17.5	10.0	26.2 ± 1.3	86.7 (1.5)	24.7 ± 1.4	85.3 (1.6)
		100.0	102.6 ± 1.9	85.1 (2.2)	102.9 ± 1.5	86.8 (1.7)
		200.0	197.2 ± 2.1	89.8 (2.3)	197.5 ± 1.2	90.7 (1.4)
Cola-1	101.2	10.0	110.1 ± 1.4	88.7 (1.6)	107.4 ± 0.9	87.3 (1.0)
		100.0	189.5 ± 1.1	88.3 (1.2)	189.2 ± 1.6	90.5 (1.8)
		200.0	280.8 ± 1.9	89.8 (2.1)	279.5 ± 1.3	90.4 (1.5)
Cola-2	60.1	10.0	68.9 ± 1.2	88.7 (1.4)	68.9 ± 2.2	88.7 (2.5)
		100.0	146.3 ± 3.0	86.2 (3.4)	148.0 ± 3.0	87.9 (3.4)
		200.0	241.9 ± 0.4	90.9 (0.4)	240.9 ± 1.4	90.4 (1.5)
Cola-3	38.3	10.0	46.8 ± 1.5	85.3 (1.7)	46.1 ± 1.8	86.3 (2.1)
		100.0	126.3 ± 2.5	88.1 (2.9)	125.3 ± 2.0	87.8 (2.2)
		200.0	224.2 ± 2.8	93.0 (3.0)	222.9 ± 1.6	92.7 (1.7)
Instant coffee-1	Not Detected	10.0	8.8 ± 0.3	88.3 (0.3)	8.8 ± 1.3	87.7 (1.5)
		100.0	89.6 ± 2.9	89.6 (3.2)	90.4 ± 0.8	90.4 (0.8)
		200.0	174.1 ± 2.5	87.1 (2.9)	181.1 ± 0.9	90.5 (1.0)
Instant coffee-2	Not Detected	10.0	9.1 ± 0.8	91.0 (0.9)	9.1 ± 0.4	90.7 (0.5)
		100.0	86.0 ± 1.8	86.0 (2.1)	91.8 ± 2.5	91.8 (2.7)
		200.0	172.6 ± 1.2	86.3 (1.4)	177.1 ± 2.4	88.5 (2.7)
Caramel-1	96.3	10.0	105.3 ± 1.0	89.7 (1.1)	106.0 ± 2.3	91.0 (2.6)
		100.0	184.4 ± 0.8	88.0 (0.9)	184.0 ± 0.9	87.2 (1.1)
		200.0	281.8 ± 1.3	92.7 (1.4)	280.4 ± 1.2	91.8 (1.3)
Caramel-2	106.7	10.0	115.4 ± 1.1	87.0 (1.2)	112.7 ± 0.5	87.0 (0.5)
		100.0	194.2 ± 2.0	87.6 (2.2)	193.2 ± 1.6	89.2 (1.8)
		200.0	292.7 ± 1.8	93.0 (1.9)	289.4 ± 1.0	92.7 (1.1)
Cola extract	116.5	10.0	125.6 ± 1.2	91.0 (1.3)	124.3 ± 1.4	87.7 (1.6)
		100.0	209.0 ± 3.1	92.5 (3.3)	207.7 ± 3.0	92.2 (3.3)
		200.0	302.0 ± 2.1	92.8 (2.3)	300.3 ± 1.5	92.4 (1.6)
Coffee-1	36.4	10.0	45.6 ± 0.9	91.3 (1.0)	44.6 ± 1.7	91.3 (1.9)
		100.0	127.3 ± 2.6	90.9 (2.9)	125.0 ± 2.0	89.5 (2.3)
		200.0	212.1 ± 1.6	87.9 (1.8)	216.1 ± 2.1	90.4 (2.3)
Coffee-2	51.8	10.0	60.0 ± 2.8	81.7 (3.4)	59.3 ± 1.4	89.0 (1.6)
		100.0	140.5 ± 2.7	88.7 (3.0)	138.8 ± 2.9	88.4 (3.3)
		200.0	225.7 ± 2.4	87.0 (2.7)	225.4 ± 1.8	87.5 (2.1)

^a^Standard deviation

### Analysis of real samples

The being applicable of the current procedures to the real samples was evaluated by resolve of 4-MeI in energy drinks, soft drinks, caramel colors, cola extract, immediate coffee and coffee samples. The actual sample analysis was carried out with the purpose of further verify the dependability and strength of the expanded process. A sum of 12 samples was examined. Resumption examinations were performed by spiking real samples with various quantities of 4-MeI (10.0, 100.0 and 200.0 µg L^−1^). In the range of 85–93%, the mean recoveries were achieved for the samples. These good improvements show that the targeted procedure is suitably appropriate to find out 4-MeI in actual samples. In all actual samples, the concentration of 4-MeI was also find out by Codex Alimentarius [49]. Spiked and non-spiked samples were provided by addition of measured volumes of working solutions of 4-MeI. The outcomes achieved by obtainable process and Codex procedures were compared employing F-test and Student’s t-test, which display that there are no important differences between outcomes achieved by two procedures at 95% confidence level (Table 4).

**Table 4:** Comparison of the results of GF-HS-SDME with the Codex method (n = 3, P = 0.95; F = 19; t = 2.78)

Sample	4-MeI added (µg L^−1^)	4-MeI found (µg L^−1^)		F-value	t-value
		GF-HS-SDME	Codex method		
Caramel- 1	0.0	107.5 ± 1.9	99.0 ± 6.7	12	2.1
	100.0	199.0 ± 4.5	204.9 ± 10.7	5.7	− 0.9
Caramel- 2	0.0	114.4 ± 5.3	110.1 ± 11.4	4.7	− 0.6
	100.0	188.2 ± 6.8	181.3 ± 2.6	7.1	1.6
Energy Drink-1	0.0	Not Detected	Not Detected	–	–
	100.0	90.6 ± 0.7	90.2 ± 1.6	5.0	0.4
Cola-1	0.0	97.7 ± 2.1	95.0 ± 1.7	1.5	1.8
	100.0	191.0 ± 1.6	188.1 ± 3.3	4.3	1.4
Instant Coffee -1	0.0	Not Detected	Not Detected	–	–
	100.0	95.9 ± 1.4	92.7 ± 2.7	3.9	1.9
Coffee-1	0.0	34.5 ± 0.8	34.0 ± 1.5	3.3	0.6
	100.0	130.2 ± 2.7	127.3 ± 1.1	6.4	1.7

**Fig. 6:**
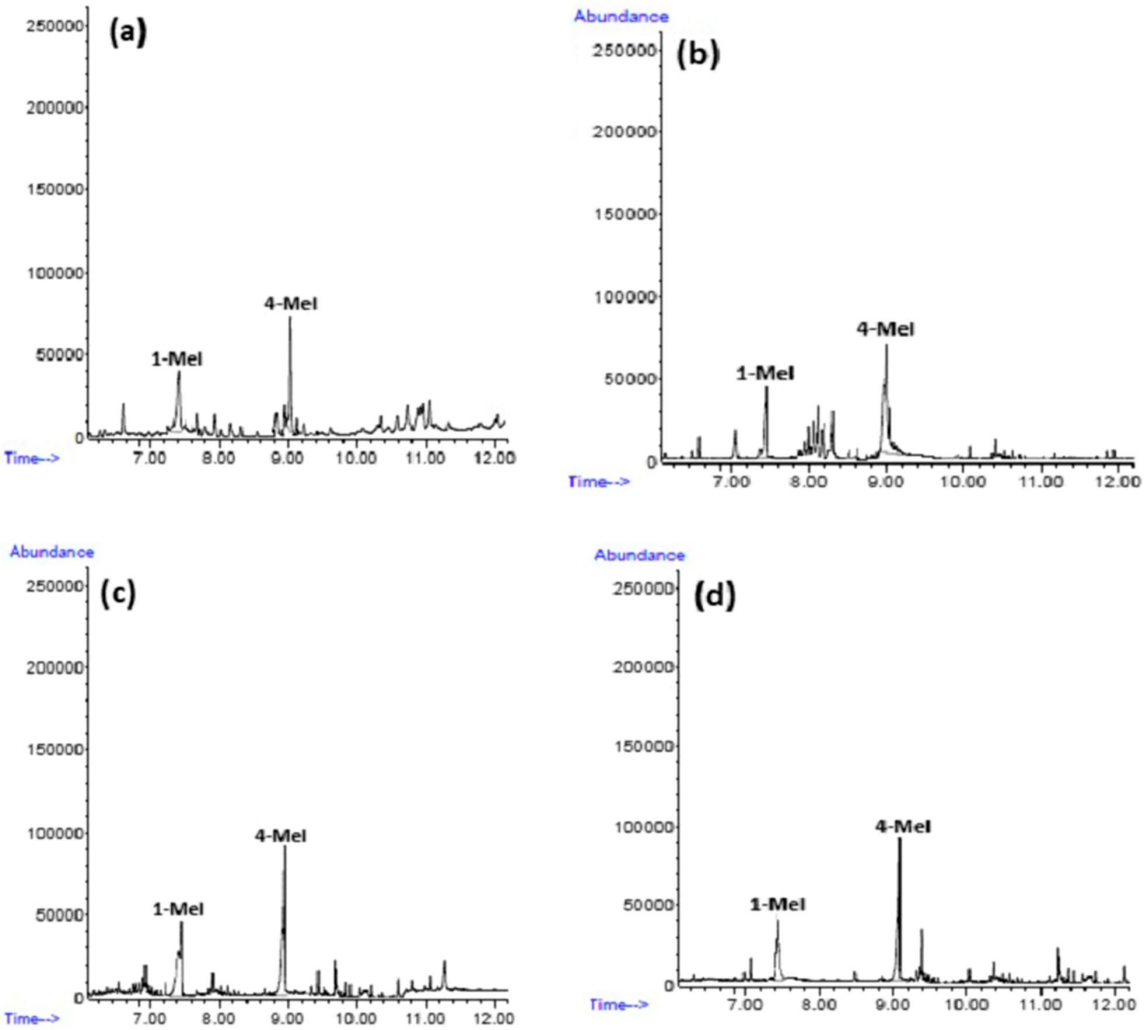
GC–MS–SIM chromatograms obtained by GF-HS-SDME of **a** caramel and **b** caramel spiked with 100.0 µg L^−1^ of 4-MeI; **c** energy drink and **d** energy drink spiked with 100.0 µg L^−1^ of 4-MeI

Figure 6 demonstrates distinctive chromatograms achieved by GF-HS-SDME GC–MS under SIM mode for spiked and unspiked energy drink and caramel samples under the selected conditions. Similar chromatograms were achieved for the other samples.

The reorganization of 4-MeI in the samples was performed by comparing the retention time (9.05 min), identifying the target (T) and qualifier (Q) ions and comparing the qualifier-to-target ratios (Q/T %) of the peaks in both the sample and the 4-MeI standard solution. The average costs for the retention time of 4-MeI pointed to very good agreement between the retention information achieved in the different samples. The goal and qualifier large quantity of materials ions were resolved by injecting individual standards under the same chromatographic conditions, except in full scan mode. The percentage of Q/T was resolved by dividing the large quantity of the selected qualifier ion by the target ion.

### The analysis of 4-MeI with other techniques for comparing of GF-HS-SDME

The yield of current GF-HS-SDME for the removal of 4-MeI and its resolving was compared by other newly reported procedures. As listed in Table 5, the procedure has minor LOD and broader linear range than most of them. Also, current procedure has a shorter linear range relative to LLE and SPE due to SDME it is not an comprehensive extraction methods.

**Table 5:** Comparison of present method with reported methods for the determination of 4-MeI

Extraction method	Instrument used	Linear range (µg L^−1^)	R^2^	LOD^a^ (µg L^−1^)	LOQ^c^ (µg L^−1^)	EF^d^	Ref
VA-IL-DLLM	UV–Vis	4–500	0.9917	1.15	3.82	200	[26]
SFE	HPLC-ESI-MS	1–70	0.9993	0.1	0.4	–	[23]
d-SPE	LC–MS	1–200	0.9996	4	10	–	[50]
ion-pair extraction	GC–MS	2000–250,000	0.9999	–	40	–	[4]
DMSPE	LC-HRMS	0.05–5 1–100	0.9994 0.9996	0.5	1	–	[22]
Dilution in eluent	LC-MS-MS	1–100	0.9998	40	80	–	[10]
d-SPE	LC-MS/MS	1–200	0.999	100	-	–	[51]
DLLME	ESIHPLC– MS–MS	0.5–500	0.999	0.1	0.3	–	[52]
Ion-pair extraction	GC–MS	250–10,000	0.9976	130	250	–	[14]
SPE	LC–MS-MS	0.3–20	0.9999	0.4	0.7	–	[20]
SPE	HPLC–ESI–MS	1–70	0.9993	0.1	0.4	–	[17]
SPME	GC–MS	6–500	0.997	1.9	6.0	–	[21]
SPE	HPCEC-PAD	50–10,000	0.997	15	45	–	[18]
Dilute-and-shoot	LC–MS TOX.IS	0–500 0–1000	0.993 0.996	1.2 ND	4.0 2.5	–	[57]
Immunization and monoclonal antibody preparation	IC-ELISA	640–20,480	0.9931	–	–	–	[25]
Conjugates of Fluorescent microspheres (FMs)	Fluorescence-based ICA	500–32,000	0.991	180	600	–	[53]
DLLME	HPLC–PDA	20–500	0.998	3.8	12.7	–	[54]
SPE	AMTC-PAD	100–10,000	0.997	15	45	–	[19]
GF-HS-SDME	GC–MS	3.2–300.0	0.998	1.1	2.9	58.5	This study

^a^Limit of detection (S/N = 3)

^b^Linear dynamic range

^c^Limit of quantification (S/N = 10)

^d^Enrichment factor

## Conclusions

In this study, a simple and green analytical method based on GF-HS-SDME was proposed for the extraction and preconcentration of 4-MeI from foodstuffs, followed by GC–MS determination. The main advantage of this method is its high preconcentration factor and fastness due to the application of an inert gas stream during microextraction. The whole analysis time was less than 20 min. The device designed for GF-HS-SDME is very simple and easy to operate and can be assembled in a few minutes. 4-MeI is a non-volatile compound which can hardly be analyzed by a HS technique; however, this method can be used for this purpose which makes a cleaner and simpler chromatogram without interferences of the non-volatile matrix of the sample. Moreover, due to the use of inert gas flow, the required temperature for extraction is lowered, so the matrix interference is significantly eliminated. As a result, this method may be usable for the analysis of thermally unstable analytes; and extraction with low boiling point solvents is achievable. Comparing with standard method of Codex, the precision of the method was higher and lower LOD and LOQ was obtained.

## Supplementary Information

**Additional file 1: Table S1.** The central composite design matrix and the experimental results.

## Data Availability

Adequate and clear descriptions of the applied materials and tools are provided in the materials and method section of manuscript. In addition, the obtained data is clearly justified by mentioning the figures and tables in the manuscript. The majority of the data used to support the findings of this study are included within the article. Other data are available from the corresponding author upon request.

## Abbreviations

ANOVA: Analysis of variance
DAD: Diode array detection
EF: Enrichment factor
GC: Gas chromatography
GC–MS: Gas chromatography-mass spectrometry
HPLC: High performance liquid chromatography
HPLC–UV: High performance liquid chromatography-ultraviolet
LOD: Detection limits
SPE: Solid phase extraction
SPME: Solid phase microextraction
UV: Ultraviolet
GLY: Glycerol
EG: Ethylene glycol
