# Supplementary material for: Application of gas flow headspace liquid phase micro extraction coupled with gas chromatography-mass spectrometry for determination of 4-methylimidazole in food samples employing experimental design optimization
Source: BMC Chem. 2022 May 6;16(1):29. doi: 10.1186/s13065-022-00823-z (PMC9077832; doi:10.1186/s13065-022-00823-z)
Supplement: Supplementary file 1 — Additional file 1: Table S1. The central composite design matrix and the experimental results. [file 13065_2022_823_MOESM1_ESM.doc]

**Additional data to:**

**Application of gas flow headspace liquid phase micro extraction coupled with gas chromatography-mass spectrometry for determination of 4-methylimidazole in food samples employing experimental design optimization**

*Mahdiye Rafiei jam[[1]](#footnote-2), Azizollah Nezhadali^1^ and Massoud Kaykhaii^2^,**

**Additional file 1: Table S1.** The central composite design matrix and the experimental results.

| Std order | **Run order** | **Pt type** | **Blocks** | **Drop volume** | **Sample volume** | **Temperature** | **pH** | **N_2_** | **GLY:EG** |
| --- | --- | --- | --- | --- | --- | --- | --- | --- | --- |
| 6 | 1 | 1 | 1 | 1.5 | 12.5 | 90.0 | 13.0 | 4.5 | 50 |
| 32 | 2 | 1 | 1 | 2.5 | 3.5 | 90.0 | 7.0 | 2.0 | 50 |
| 8 | 3 | 1 | 1 | 1.5 | 12.5 | 30.0 | 7.0 | 4.5 | 50 |
| 47 | 4 | 0 | 1 | 2.0 | 8.0 | 60.0 | 10.0 | 3.25 | 60 |
| 4 | 5 | 1 | 1 | 2.0 | 8.0 | 30.0 | 10.0 | 3.25 | 60 |
| 26 | 6 | 1 | 1 | 2.0 | 8.0 | 60.0 | 10.0 | 3.25 | 60 |
| 10 | 7 | 1 | 1 | 1.5 | 12.5 | 30.0 | 13.0 | 2.0 | 50 |
| 37 | 8 | -1 | 1 | 1.5 | 12.5 | 90.0 | 13.0 | 2.0 | 70 |
| 49 | 9 | 0 | 1 | 2.5 | 12.5 | 90.0 | 7.0 | 4.5 | 50 |
| 48 | 10 | 0 | 1 | 1.5 | 3.5 | 90.0 | 13.0 | 2.0 | 50 |
| 53 | 11 | 0 | 1 | 2.5 | 3.5 | 30.0 | 13.0 | 2.0 | 50 |
| 31 | 12 | 1 | 1 | 2.0 | 12.5 | 60.0 | 10.0 | 3.25 | 60 |
| 30 | 13 | 1 | 1 | 2.5 | 3.5 | 30.0 | 7.0 | 2.0 | 70 |
| 42 | 14 | -1 | 1 | 2.5 | 12.5 | 90.0 | 13.0 | 4.5 | 70 |
| 52 | 15 | 0 | 1 | 2.0 | 8.0 | 60.0 | 10.0 | 3.25 | 70 |
| 40 | 16 | -1 | 1 | 2.0 | 8.0 | 90.0 | 10.0 | 3.25 | 60 |
| 44 | 17 | -1 | 1 | 2.5 | 12.5 | 30.0 | 7.0 | 2.0 | 50 |
| 22 | 18 | 1 | 1 | 2.5 | 3.5 | 90.0 | 7.0 | 4.5 | 70 |
| 23 | 19 | 1 | 1 | 2.5 | 3.5 | 90.0 | 13.0 | 4.5 | 50 |
| 9 | 20 | 1 | 1 | 2.0 | 8.0 | 60.0 | 7.0 | 3.25 | 60 |
| 5 | 21 | 1 | 1 | 2.5 | 12.5 | 30.0 | 13.0 | 2.0 | 70 |
| 50 | 22 | 0 | 1 | 1.5 | 3.5 | 30.0 | 13.0 | 4.5 | 50 |
| 12 | 23 | 1 | 1 | 1.5 | 3.5 | 90.0 | 7.0 | 4.5 | 50 |
| 39 | 24 | -1 | 1 | 1.5 | 3.5 | 30.0 | 7.0 | 2.0 | 50 |
| 41 | 25 | -1 | 1 | 1.5 | 8.0 | 60.0 | 10.0 | 3.25 | 60 |
| 24 | 26 | 1 | 1 | 1.5 | 3.5 | 30.0 | 13.0 | 2.0 | 70 |
| 51 | 27 | 0 | 1 | 2.5 | 8.0 | 60.0 | 10.0 | 3.25 | 60 |
| 38 | 28 | -1 | 1 | 2.0 | 8.0 | 60.0 | 10.0 | 4.5 | 60 |
| 35 | 29 | -1 | 1 | 2.5 | 3.5 | 30.0 | 13.0 | 4.5 | 70 |
| 46 | 30 | 0 | 1 | 1.5 | 3.5 | 90.0 | 13.0 | 4.5 | 70 |
| 43 | 31 | -1 | 1 | 1.5 | 3.5 | 90.0 | 7.0 | 2.0 | 70 |
| 28 | 32 | 1 | 1 | 2.5 | 3.5 | 90.0 | 13.0 | 2.0 | 70 |
| 3 | 33 | 1 | 1 | 1.5 | 12.5 | 30.0 | 7.0 | 2.0 | 70 |
| 18 | 34 | 1 | 1 | 2.0 | 3.5 | 60.0 | 10.0 | 3.25 | 60 |
| 17 | 35 | 1 | 1 | 2.0 | 8.0 | 60.0 | 10.0 | 3.25 | 60 |
| 20 | 36 | 1 | 1 | 1.5 | 12.5 | 90.0 | 7.0 | 4.5 | 70 |
| 34 | 37 | -1 | 1 | 1.5 | 3.5 | 30.0 | 7.0 | 4.5 | 70 |
| 19 | 38 | 1 | 1 | 2.0 | 8.0 | 60.0 | 10.0 | 2.0 | 60 |
| 11 | 39 | 1 | 1 | 2.0 | 8.0 | 60.0 | 10.0 | 3.25 | 60 |
| 7 | 40 | 1 | 1 | 2.0 | 8.0 | 60.0 | 10.0 | 3.25 | 60 |
| 21 | 41 | 1 | 1 | 1.5 | 12.5 | 90.0 | 7.0 | 2.0 | 50 |
| 13 | 42 | 1 | 1 | 2.5 | 3.5 | 30.0 | 7.0 | 4.5 | 50 |
| 29 | 43 | 1 | 1 | 2.0 | 8.0 | 60.0 | 10.0 | 3.25 | 60 |
| 1 | 44 | 1 | 1 | 2.5 | 12.5 | 30.0 | 13.0 | 4.5 | 50 |
| 33 | 45 | -1 | 1 | 2.0 | 8.0 | 60.0 | 10.0 | 3.25 | 60 |
| 2 | 46 | 1 | 1 | 2.5 | 12.5 | 30.0 | 7.0 | 4.5 | 70 |
| 16 | 47 | 1 | 1 | 2.0 | 8.0 | 60.0 | 10.0 | 3.25 | 60 |
| 45 | 48 | 0 | 1 | 2.0 | 8.0 | 60.0 | 10.0 | 3.25 | 50 |
| 27 | 49 | 1 | 1 | 2.5 | 12.5 | 90.0 | 7.0 | 2.0 | 70 |
| 25 | 50 | 1 | 1 | 2.0 | 8.0 | 60.0 | 13.0 | 3.25 | 60 |
| 14 | 51 | 1 | 1 | 2.5 | 12.5 | 90.0 | 13.0 | 2.0 | 50 |
| 36 | 52 | -1 | 1 | 2.0 | 8.0 | 60.0 | 10.0 | 3.25 | 60 |
| 15 | 53 | 1 | 1 | 1.5 | 12.5 | 30.0 | 13.0 | 4.5 | 70 |

1. ^*^Corresponding author. Tel: +48731960312; E-mail: kaykhaii@gmail.com

   ^1^Department of Chemistry, Payame Noor University, P.O. Box 19395–4697, 19569 Tehran, Iran

   ^2^Department of Process Engineering and Chemical Technology, Faculty of Chemistry, Gdansk University of Technology, Gdansk, Poland [↑](#footnote-ref-2)
